# Patient and Public Involvement and Engagement (PPIE) in methodological research: exploring challenges, tensions and learning from the perspectives of PPIE and researchers in the Realist Economic Evaluation Methods (REEM) study

**DOI:** 10.1186/s40900-026-00956-8

**Published:** 2026-09-04

**Authors:** Andrew Fletcher, Violet Rook, Susan Mountain, Felicity Shenton, Linda Fenocchi, Meghan Bruce Kumar, Sonia Dalkin, Angela Bate, Rebecca Hunter

**Affiliations:** 1 University of Northumbria, Newcastle upon Tyne, UK; 2 Newcastle upon Tyne, UK; 3 National Institute for Health and Care Research (NIHR), Applied Research Collaboration North East and North Cumbria, Cumbria, Northumberland, Tyne & Wear NHS Foundation Trust, Newcastle upon Tyne, UK; 4 Glasgow Caledonian University, Glasgow, UK

**Keywords:** Methodological development, Methodology research, Methodological research participation, Co-production, PPIE, Community engagement, Health and social care research

## Abstract

**Background:**

Methodological research is often abstract, heavily theoretical and jargon laden. Despite having significant implications for the users of health and social care services, it can be difficult to involve the public and patients in research that is oriented towards more academic ‘ways of knowing’. This article combines Public and Patient Involvement and Engagement (PPIE) and university researcher perspectives on their experiences working on a study that aimed to develop a new evaluation approach.

**Methods:**

Using a co-produced reflective case study design, four university researchers and three (representing a wider group of six) PPIE contributors reflected on their shared experiences of PPIE within the Realist Economic Evaluation Methods (REEM) methodological study. Reflections were gathered during a structured two-hour workshop, which identified three broad initial themes. These were explored in depth by academic/PPIE writing duos across two online follow-up meetings, producing collaborative written summaries. A fourth academic synthesised the summaries to ensure coherence and the final manuscript was examined and approved by the full team.

**Results:**

The challenges encountered during the study were organised into three broad themes: (1) Identifying appropriate PPIE contributors; (2) maintaining and sustaining engagement; and (3) determining when and how best to engage.

**Conclusion:**

We propose that PPIE in methodological health and social care research requires different considerations than PPIE in applied research, including actions to normalise uncertainty and be creative about modes of contribution as part of co-learning. We have articulated these as recommendations, with implications for funders/commissioners of research, university researchers and PPIE contributors alike.

## Abstract (plain language summary)

Patient and Public Involvement and Engagement (PPIE) is critical in making health and care research meaningful and relevant for those it seeks to help. Implementing PPIE in applied research, specific to a particular health condition, social issue or population, can be more straightforward compared with implementing PPIE in more abstract, theoretical or methodological research (i.e., research that focuses on developing, testing and refining the tools, techniques and approaches that other researchers use). This article reports the experiences of PPIE contributors and university researchers working together on one such study, which combined two different methodological approaches. A workshop was held towards the end of the study to gather the team’s reflections on working on a project where the public beneficiaries were not immediately clear. Challenges included, (1) how to identify the most suitable PPIE contributors for such a study; (2) maintaining and sustaining their engagement; and (3) developing methods and activities that would enable PPIE to contribute most meaningfully, beyond tokenistic engagement. We conclude that PPIE in methodological research is possible but must be considered different from ‘standard’ PPIE practices in applied research, requiring careful planning by research teams. To this end, we have compiled some relevant considerations and recommendations – potentially the beginnings of a more formal framework – to help methodological research teams and PPIE contributors work better together.

## Background

Guidance on meaningful Patient and Public Involvement and Engagement (PPIE) in methodological (as opposed to applied) research is scant. This article draws on a reflective case study that includes both PPIE contributors’ and researchers’ experiences trying to achieve this in a highly theoretical methodological development project.

A fundamental principle of meaningful health and social care research is ensuring that the voices of those who use, deliver, and are affected by services are heard and help to shape the research itself. The National Institute for Health and Care Research (NIHR) claimed, over a decade ago, that public involvement in research should be “second nature” by 2025 [1, 2] and now mandates PPIE, with a named PPIE lead across all its funded research programmes [3]. PPIE is distinct from participatory research methods more broadly: although the two share some commonalities, they differ in their decision-making dynamics, their intended impact on the research, and the partners they involve, with PPIE specifically representing patient and public perspectives rather than a wider set of stakeholders [4]. While PPIE is increasingly evident in applied health and social care research, its implementation is neither universal nor straightforward. And despite the existence of PPIE frameworks and guidance [5], PPIE is subject to variable practices and inconsistencies in research design, contributor involvement and decision making. For example, there are conflicting assumptions about the purpose and value of involvement: some approaches are pragmatic and outcome-oriented, focused on improving research quality, while others are ideological and rights-based, emphasising democratic representation, accountability, and the redressing of power imbalances [6]. Similarly, efforts to ensure PPIE contributions are meaningful and not tokenistic can also complicate the design, delivery and decision-making processes in research projects [5, 7].

In the United Kingdom (UK), guidance for PPIE in research has largely been developed from practices emerging in applied health and social care research. The Care Act [8] encourages and provides a framework to support PPIE in research but does not mandate it outright. Nevertheless, most publicly funded health and social care research must now evidence the involvement of patient and public participants. The Social Care Institute for Excellence [9] describes six principles of co-production in research: building on people’s existing capabilities; promoting mutuality and reciprocity; developing peer support networks; breaking down boundaries; facilitating as well as delivering; and recognising people and their experiences as assets. PPIE roles bring lived experience to the heart of the research process by helping to frame research questions and ensuring studies serve the people they are meant to help [10, 11]. However, the current PPIE frameworks and guidance focus on applied (health) research, with little distinction of the role of PPIE in methodological research and the resulting challenges and tensions. While applied research uses data to “help solve a real-life problem” [12], methodological research aims to improve research methods and knowledge about the research process itself, with methods as the subject of study (e.g., developing, refining, or comparing approaches to data collection, analysis, or study design) [13]. This makes methodological research more theoretical and further removed from patient and public experiences, meaning PPIE contributors may struggle to see how their input is relevant [14].

This challenge is reflected in a small but growing literature exploring PPIE roles in ‘less applied’ research. Studies in informatics and big data research have highlighted how the abstract nature of the work creates particular demands on PPIE contributors, including reliance on trust in researchers and inconsistency in how involvement is defined and enacted [15, 16]. Some researchers have questioned whether highly technical areas can support meaningful public involvement at all [17]. The issue has been observed in implementation research [4] and is especially true in statistical methodology research [18], resulting in the formation of the *Patient and Public Involvement for Statistical Methodology and Research Techniques* (PPI-SMART) group, which aims to bridge the gap between public contributors and statistical methodologists [19]. PPI-SMART has itself asked, “when it comes to highly technical statistical methodology research, is patient and public involvement and engagement relevant, or just a funding application tick-box exercise?” [20]. Recognition of these challenges is growing at a policy level too; the Medical Research Council’s one-off funding call for ‘Understanding public involvement in non-clinical research’ [21] explicitly included methodological research as a focus area, acknowledging that approaches developed in applied research contexts may not translate well to more theoretical-focussed studies.

This was exemplified in a research project, undertaken by the authors, which focused on developing and testing a new methodological approach rather than generating findings about a health condition or population. Our NIHR-funded Realist Economic Evaluation Methods (REEM) study (NIHR135102) is an example of methodological research that had PPIE embedded throughout. REEM aimed to develop a new approach for evaluating complex interventions by integrating methods from realist evaluation and health economic evaluation, and producing guidance and a graphic summary for their application [22, 23]. Existing PPIE approaches did not straightforwardly translate to the methodological research, resulting in specific challenges for both the university researchers and the PPIE contributors. This article uses REEM as a case study to describe our approach to PPIE and to explore some of the practical application, challenges and tensions we encountered in implementing PPIE in methodological research.

### Aims of the case study

We start from the premise that systematic PPIE is always important in health and care research [24], but that different study types – in this case, methodologically-focussed research – bring about different challenges and considerations. These are explored here through a single reflective case study, to provide lessons and strategies to support meaningful PPIE in methodological research.

This article, co-authored with the REEM PPIE team, is intended as a commentary rather than a report of a formal empirical study, and as such does not follow a traditional research methodology. However, we have adopted section headings (background, design and methods, findings, and discussion) purely as an organisational device, to help structure our reasoning and present our argument in a clear and logical sequence. Accordingly, this section describes our approach to developing the ideas presented in this piece, rather than a set of methods in the conventional research sense.

## Design and methods

This was a co-produced reflective case study design, taking REEM – a methodological research study – as the case [1]. PPIE contributors and university researchers reflected on their shared experiences of incorporating meaningful PPIE in REEM, and these reflections constitute the data [2]. The team generated these reflections specifically for this article through a workshop and writing process, rather than drawing on records or outputs from the REEM project itself.

### The case

**The REEM study** sought to combine methods used in realist evaluation and economic evaluation, to develop a new approach in evaluating complex health and care interventions, and to develop guidance for researchers using this new approach. The project had three stages: (1) exploring the methodology literature to determine theoretical similarities and differences between the two approaches; (2) running three ‘pilot’ studies to explore possible ways to combine the approaches; and (3) developing guidance based around learning from stages one and two. PPIE involvement was embedded across all three phases.

The PPIE group comprised six contributors: a co-applicant who also co-chaired the PPIE group, a PPIE coordinator, and four additional members with substantial prior PPIE experience. The coordinator was a non-academic role, but led the PPIE team, facilitated involvement and acted as a bridge with the university researchers. The REEM PPIE team brought adjacent knowledge, for example, being experts by experience as PPIE contributors to evaluation research and/or funder panels. The research team sought to uphold principles of good PPIE [25] throughout, using co-production and shared decision making wherever possible [26].

PPIE contributions were structured around five key activities: [1] developing understanding of realist and economic evaluation and the benefits and challenges of combining the two approaches; [2] reviewing methodological guidance from a PPIE perspective; [3] reviewing the pilot evaluation studies to understand their methodological and PPIE implications; [4] contributing to dissemination of learning through lectures, seminars, and opinion pieces; and [5] reviewing final REEM guidance and developing lay summary materials to support future involvement. The PPIE co-chair and coordinator attended monthly study team meetings in addition to the regular PPIE workshops and group sessions, where contributions and challenges were discussed. While some parts of the project had more PPIE-relevance than others, PPIE contributors were kept informed of every stage and all major decisions via email and monthly meetings. They could choose to engage at any point, so actively navigated and determined their own contribution. ‘Depth of involvement’ was therefore an ongoing negotiation rather than predetermined.

### Approaches to data generation

For the reflective case study presented, data were generated through a structured two-hour online workshop and two online follow-up writing sessions. The workshop comprised three PPIE contributors and four university researchers.^1^ All were invited to provide examples of, and their reflections on, challenges of PPIE in methodological development research, implications for PPIE involvement in the context of research project ebb and flow, and whether and how the duty of care for PPIE in methodological research is different from empirical research.

All group members brought specific issues and examples of when PPIE had been successful during the REEM project and when it had been unsuccessful. The group reflected on these experiences, exploring how they might have been avoided or overcome. These were organised into three broad themes towards the end of the workshop and agreed by all members. The themes were: (1) identifying appropriate PPIE contributors; (2) maintaining and sustaining engagement; and (3) when and how to engage.

Following the workshop, three writing duos, each comprising one academic author and one PPIE contributor, were formed. Each duo focused on a theme and collaboratively produced a short (approximately 1,000-word) summary, including loose recommendations. All three summaries were co-written, reflecting the perspectives of each duo and the wider group. A fourth academic reviewed all summaries to ensure that the perspectives discussed in the workshop were fairly represented and preserved, and marshalled the themes and recommendations into a draft article. Drafts of the manuscript were subsequently edited and discussed via email and in two online meetings with all co-authors. The processes for creating this article were done in accordance with the NIHR principles of co-production [26] to ensure all voices had equal status. All co-authors viewed and approved the final draft. Ethical approval for the REEM study was obtained from the Faculty Research Ethics Committee at Northumbria University (Project #1673) on 25 January 2023.

## Findings

Three themes were identified through the workshop and online meetings: (1) identifying appropriate PPIE contributors; (2) sustaining interest and engagement; and (3) determining when and how to engage. The following sections present edited versions of the co-produced written material described above.

### Identifying appropriate PPIE contributors in methodological research

One of the challenges of understanding the value of PPIE in methodological development is first clarifying *who* are the appropriate patients and public to involve. Unlike applied research, where lived experience of a condition is often the defining expertise, methodological research has no specific patient group at its centre. Instead, the defining expertise is exposure to research design and methodological decisions, and the ability to reflect on that exposure, which is often built on prior involvement as a PPIE contributor to empirical research. Teams must therefore think more broadly about who can contribute their own understanding of a problem and be explicit about this when recruiting. The ultimate beneficiaries of methodological research are still patients and the public – better research methods should contribute to better health outcomes – but this relationship is indirect. PPIE contributors must therefore be selected and supported with this in mind, representing the interests of those who will ultimately benefit, even if that benefit is one step removed from the research itself. Despite being health and social care-oriented, the need for the REEM PPIE group to recognise methodology as an end in itself (rather than as a means to benefit particular groups) was prioritised. Consequently, more focus was placed on the ‘public’ than the ‘patient’ element of PPIE, and we specifically recruited those with experience of being involved in range of health and social care evaluations and/or as PPIE reviewers of research on funding panels.

To be relevant and impactful beyond academia, the outputs of methodological research must connect with a dynamic and complex set of audiences. PPIE contributors therefore need to think beyond individual contexts and consider the roles and implications of methodological research in broader health and care structures. This may sit alongside a broader engagement strategy; involving funders or commissioners of services, practitioners who might use the research, or generators of evidence. However, it remains important to maintain a distinction between this broader stakeholder engagement and PPIE itself; PPIE retains a specific function in representing the interests of patients and the public. In REEM, this required careful consideration of positionality, particularly in relation to commercial determinants of health and the private interests some stakeholders might hold, including around commercialising data and other research outputs that could lead to undue influence on outcomes.

### Maintaining and sustaining engagement in methodological research

Methodological research often progresses unevenly, with periods of intense development interspersed with longer stretches of background work or reflection. During these quieter phases, maintaining interest and momentum among PPIE contributors can be challenging. In REEM, for example, the extensive literature scoping stage reduced the immediacy of PPIE activity, disrupting the sense of continuity, shared purpose and connection with the study’s overall aims. While refresher updates and summaries were provided, contributors frequently needed clarification on how their lived experience could inform methodological decisions. Even with structured support, this issue highlighted the unique challenge of sustaining engagement and a sense of impact in highly conceptual research.

Both the university researchers and the PPIE contributors found the exploratory and abstract nature of the work cognitively challenging. Research involving PPIE usually uses established methods and tools, which most team members have some familiarity with. The REEM project was engaged in *creating* a tool – a guidance document for reconciling two different research paradigms. These draft principles and guidance were developed as the study progressed, meaning those in a PPIE role needed support to engage with complex evolving ideas that were being iteratively tested and formalised. One PPIE contributor noted the difficulty in visualising the end point of methodological research, compared with empirical [applied] research, where end point, product or benefit is often clearer. This speaks to the nebulous nature of methodological research; where the destination exists but the path towards it remains unclear for much of the journey, making it difficult for PPIE contributors to locate their contribution within the wider work.

This uncertainty became apparent at an early stage in REEM. Structured support and responsive encouragement were provided through regular self-directed meetings, which focussed on small specific tasks, breaking down the cognitive challenge into more manageable chunks. For example, exploring small sections of the draft guidance to discuss potential PPIE implications. While this approach helped to maintain engagement, some tasks felt disconnected from the overarching project. In these moments, where their contribution to a specific task was less clear, some PPIE contributors felt both disengaged and frustrated. One commented, *“there is a risk of always feeling that if I am not putting something into the meeting that day then you are no good to them. But I learned that in some meetings I won’t contribute and in some I will give nuggets [of contribution]”*. With time and experience it became possible for PPIE contributors to feel more comfortable navigating the possibilities for offering contributions. In addition, the uncharted nature of the work also had its benefits; precisely because nobody knew the territory, researchers and PPIE contributors alike found themselves navigating unfamiliar ground together, creating a sense of ‘togetherness’ that helped to reduce feelings of hierarchy and sustain engagement. As one PPIE contributor reflected during the planning workshop for this article: *“since no-one knew the road ahead*,* that did help to feel like we were all in it together”*.

### When and how to engage

Methodological research can lack predictability in comparison to more applied projects, making it difficult to identify when and how PPIE contributions would be most meaningful. The PPIE contributors in REEM were eager to engage throughout, but not every stage offered clear entry points, particularly when discussions concerned more technical or theoretical detail. As noted above, this sometimes left the contributors uncertain about their role and how their input would be applied. In the same circumstances, the academic team sometimes deemed PPIE input unnecessary for certain methodological decisions. While this may seem exclusionary, it highlights the challenge of balancing expert and public perspectives in conceptual work. As a result, contributors sometimes experienced a lack of reciprocal dialogue with the university-based researchers. It also raised broader questions about *who* should determine the timing and extent of involvement and how resources such as time and funding, could be best used to enable the most meaningful engagement.

The balance of when and how to involve contributors was not always right. There were times when decisions about timing and scope of PPIE input were made too narrowly or too late in the process. While imperfect, these moments offered valuable opportunities to reflect on how future methodological projects could approach engagement differently, particularly by involving contributors earlier, to address questions of when and how to engage from the outset. An example of these dynamics was the development of the guidance itself. The project team drafted initial versions of the guidance and invited the PPIE group to provide feedback, which informed subsequent refinements. Contributors indicated that they would have welcomed opportunities to be involved earlier in shaping the guidance.

The challenges and learning across these three themes are summarised alongside key recommendations in Table 1.

## Discussion

Standard PPIE in applied health and social care research typically makes clear contributions through well-defined roles that draw on the lived experiences of end users and beneficiaries. In methodological research, study designs are often focussed on what takes place ‘behind the scenes’, in the more abstract or technical spaces usually hidden from public view, where the role of PPIE is less clear. This uncertainty echoes questions raised by Abell et al. [17] about whether highly technical research can support meaningful involvement at all. In the REEM study, this ambiguity surfaced practical and conceptual challenges around defining contributions, managing cognitive and technical complexity, and balancing iterative methodological development with meaningful outcomes-oriented involvement. A similar pattern of challenges – around preparation, sustaining involvement and selecting suitable tasks – has been reported in health economics methods research specifically [14], suggesting these are not unique to REEM.

‘Conventional’ PPIE is not a fixed or universally agreed standard. Terminology, models and expectations vary between applied and methodological research, and across countries, funders and disciplines; even well-established terms such as ‘stakeholder’ are contested, with no international consensus on alternatives [27]. Recent examples from the wider literature illustrate this diversity: in the United States, the study by Smith et al., on community-based participatory research frames involvement through “partners” and epistemic authority rather than standard PPIE terminology [28], while in Germany, visual methods such as the ‘Gallery Walk’ have been used to involve people with dementia in ways that depart from committee- or workshop-based models [29]. Our own institution, [university name redacted] in the UK, has recently designed a course on PPI and co-production, which accounts for a broad range of involvement contexts. Rather than drifting towards academic ‘niche-ification’, we believe this reflects a growing awareness of the need for PPIE, and so an accompanying requirement for deeper understanding and more targeted ways to engage. Set against this backdrop, we see our approach in REEM less as a deviation from ‘conventional’ PPIE and more as a tailored response to the specific demands of a particular research context. The experiences described here not only highlight some of the challenges we encountered, but also reinforce the need for multiple ways to approach PPIE, and a corresponding need for better mapping and guidance for different types of research.

Choices about which PPIE contributors to involve and the nature of their contributions are to be expected, but in REEM these raised broader questions about how decisions are made and the level of engagement expected at different stages of the study. With no formal guidance available on PPIE in methodological research, the research team relied on expectations derived from PPIE in applied research, which were not always transferable. This gap reflects a broader issue: the MRC has itself acknowledged that PPIE methods built for applied contexts do not always fit more conceptual or theoretical work [21], and our experience in REEM offers a practical illustration of this. As Brett et al. [30] note, such situations can give rise to challenges when service users are involved sporadically or without a clear role. Comparable debates appear in other technical domains: participatory software development, for example, has long wrestled with balancing co-design and technical integrity [31], while collaborative approaches in environmental modelling caution against token or nominal stakeholder inputs when engagement is not meaningfully integrated [32]. These parallels suggest that methodological research in health and social care faces similar challenges to other abstract / technical fields that depend on expert-lay collaboration.

These challenges are further complicated by the diversity of PPIE contributors. The collective term PPIE does not represent a homogenous group [33] and contributors may hold differing views on the level of engagement necessary. In REEM, some contributors preferred their engagement to ‘ebb and flow’ according to study phases; they were happy to have deeper involvement in elements of the project that they felt were more relevant to their needs (or the needs of other end users). Others wanted involvement in all decisions, even technical ones; they believed that involvement must be comprehensive otherwise it risked being controlled by researchers, resulting in a power imbalance. This illustrated a dichotomy between optimal and maximal participation, with corresponding implications for balancing efficiency against equality. Failure to consider and to carefully manage these factors across all aspects of the research plan may risk compromising academic rigour [30, 34]. This tension was observed in REEM and, in the moment, required careful reflection to support meaningful contributions without undermining study integrity.

A further practical challenge in methodological research is cognitive burden. It was felt strongly throughout the REEM project that while methodological research can be made accessible if communicated clearly, the PPIE contributors had a distinct role and did not need to master all the technical details. Nevertheless, they were supported with explanatory materials and workshops outlining the basics of realist evaluation and economic evaluation, and the relevance to health services of combining these. This helped them understand the purpose and the parameters of the study, but they did not have – nor need – the depth of knowledge required to fully engage with the theoretical aspects of realist evaluation and economic evaluation. In addition, the resources to do this would have been far beyond the scope of this study (and, we suspect, most other studies).

Accounting for the additional cognitive burden created by managing unfamiliar information has been reported elsewhere. For example, in evaluating the ‘quality’ (inverted commas in original) of their own approach to PPIE in a conceptual study, Lammons, Nobility [35] recognised that sharing various types of new information generated “cognitive burden” [35] and sought to reduce this by amending their internal evaluation approach. Similar issues can be seen in other disciplines where stakeholders contribute to conceptual or technical guidance. For instance, participation in abstract or technical policymaking [36]; participatory approaches in design research [37]; and co-production in environmental and climate modelling [38] have all highlighted tensions around expertise, accessibility and maintaining engagement with abstract material. Across these fields, successful approaches emphasise iterative communication, flexible participation, and a shared tolerance for uncertainty – principles that resonate strongly with our own learning from REEM.

In our study, contributors reported aspects of cognitive burden related to the abstract nature of the work, but this did not hinder their engagement; rather, it fostered shared reflection and collaborative learning across the team. In addition, Liabo, Asare [39] note that in research not focused on specific disease areas or health and social conditions, some familiar risks, such as emotional burden, are less pronounced. These experiences highlight the importance of anticipating and mitigating both emotional and cognitive risks for PPIE in methodological research, particularly during more abstract or technical phases.

Finally, these reflections underscore the need to balance meaningful PPIE involvement with methodological rigour. In comparison to applied research, where a lack of rigour may generate findings that are less useful (or potentially harmful) to the research beneficiaries, methodological research that lacks rigour is more likely to harm research itself, potentially ultimately eroding public trust in science. Involving PPIE in the development of new methodologies therefore requires careful management to prevent unintentionally compromising academic standards or established principles.

### Recommendations

Based on the challenges and opportunities for learning articulated throughout this manuscript, it is clear that different types of research demand distinct approaches to PPIE, underpinned by appropriate frameworks and guidance. The recommendations summarised in Table 1 provide an initial foundation for supporting meaningful involvement in methodological research. To ensure consistency and quality across future studies, a dedicated framework tailored specifically to more abstract and theoretical forms of research would be a valuable next step.

**Table 1:** Recommendations for PPIE in methodology-focussed research

Theme	Example from REEM study	Learning from this, plus key considerations and actions
Theme 1: Identifying appropriate contributors	PPIE contributors were experienced in multiple types of research, but were less familiar with methodological work, including how and by whom it would be used. The link with the end beneficiary was less clear.	**Define PPIE broadly and intentionally**. Move beyond condition- or disease-specific expertise to include contributors who can think conceptually and represent wider public perspectives. Be explicit about what is needed, why they are involved, and what kind of contribution is sought. Attend to positionality and potential conflicts of interest.
Theme 2: Maintaining and sustaining engagement	There were periods during which the PPIE team felt disengaged or, when they were involved, were unclear about how they could contribute. For some, this was demotivating.	**Build flexibility and responsive support**. Recognise that engagement and contributors’ sense of impact will fluctuate. Provide tailored support, including relevant training, focused task-based meetings and opportunities for reflection, beyond standard activities. **Create continuity through communication**. Maintain connection with contributors during quieter or highly technical phases through regular updates, check-ins, and clear explanations of how their input links to the ongoing work.
Theme 2: Maintaining and sustaining engagement	The university-based team were also sometimes unsure about directions and outcomes, which was difficult to convey to PPIE.	**Normalise uncertainty as part of co-learning**. Acknowledge that methodological work is iterative and sometimes ambiguous. Encourage both researchers and contributors to view uncertainty as an opportunity for shared learning.
Theme 2: Maintaining and sustaining engagement	Some PPIE contributors found REEM confusing. The project had to be sensitive to their needs, while also upholding rigorous academic standards.	**Anticipate and mitigate methodological-specific risks**. Recognise distinct risks, such as cognitive burden for contributors or threats to methodological rigour. Use pacing, plain-language explanations, and mutual reflection to maintain inclusivity and scientific integrity.
Theme 3: When and how to engage	Users of REEM outputs would not generally be patients / public, so the project had to devise new forms of meaningful involvement.	**Be creative about modes of contribution**. When conventional PPIE activities (e.g. reviewing materials, sense-checking outputs) are less relevant, design new forms of contribution such as concept testing, or exploring real-world implications.
Theme 3: When and how to engage	Researchers and PPIE contributors alike realised that REEM had been different from their usual experience, requiring deeper enquiry.	**Reflect**,** adapt and share learning**. Treat each methodological project as a learning opportunity. Build in reflective spaces for researchers and contributors to identify what worked and what didn’t and share this learning to inform evolving PPIE frameworks.

## Strengths and limitations

Our approach to generating findings for this article meant that researcher and PPIE contributor perspectives were represented and balanced throughout, from data generation to manuscript development. The reflective process was guided by the PPIE team and responded to by the university-based team, which all contributors felt was valuable and lent authenticity to the findings. The richness of the reflective case study approach allowed for honest, nuanced accounts of what PPIE in methodological research looks and feels like in practice, and the recommendations are grounded in the findings and lived experiences captured through engagement with a real-world study.

There are also important limitations. This is a single reflective case study drawing on a small purposive sample, and findings should be understood as reflections from one project rather than generalisable conclusions. The reflective design means that data are retrospective and subject to recall, and the perspectives of the full PPIE group of six are represented through three co-authors. The PPIE contributors in REEM were recruited for their experience of involvement in health and social care evaluations and on research funding panels, rather than lived experience of specific health conditions. This profile differs from condition-focused PPIE, so findings may not transfer directly to studies involving contributors with that more traditional profile. Finally, REEM focused on a specific type of methodological research; teams working in different methodological contexts may face different challenges.

We remind readers that although this article is submitted as a commentary rather than a formal research study, the structured approach taken in our reflective process, data generation and reporting, was of significant benefit in organising our argument. The core message remains cohesive, valid and important; that PPIE in methodological-oriented research differs from PPIE in more applied research, so requires additional consideration.

## Conclusion

Approaches to PPIE developed in applied research do not transfer straightforwardly to methodological, abstract, iterative, or more technical/theoretical studies. This was apparent in the unique challenges we observed in the REEM study, including: defining contributions, balancing involvement with methodological rigour, managing cognitive burden, maintaining interest/engagement and pacing PPIE activities. These issues highlight that existing guidance on PPIE does not address the full gamut of research contexts. Tailored guidance for PPIE in methodological-focussed research could help research teams identify *who* to involve, *when* contributions are most valuable, and *how* abstract discussions can be made meaningful, while also supporting contributors to sustain engagement and see the impact of their input.

While this article reflects only our experience, in a single reflective case study, we see a need for further discussion of public involvement in methodological research, with the potential even for developing a new term, with specific guidance. Our experiences in REEM surfaced challenges and tensions that prompted the development of practical strategies, shared here as observations and recommendations. These insights offer a preliminary framework for embedding meaningful PPIE in methodological research, and a foundation on which researchers, contributors, and funders can together build more robust and inclusive practice. Given that PPIE is mandated by most major health and social care research funders, we suggest that funding organisations draw on insights such as those presented here to develop tailored PPIE guidance and requirements for methodological research. Closer dialogue between funders, methodologists and public contributors would help ensure that PPIE is both rigorous and achievable across the full spectrum of funded research.

## Data Availability

No datasets were generated or analysed during the current study.

## Footnotes

1 All six members of the REEM PPIE group were invited to co-author this paper. The three who participated did so based on their personal availability and inclination to take on the role. This highlights a reality of co-produced research: participation often depends on the varying capacity and time of public contributors.

## Abbreviations

PPIE: Patient and Public Involvement and Engagement
REEM: Realist Economic Evaluation Methods
NIHR: National Institute for Health and Care Research
UK: United Kingdom
